# Transesophageal and intracardiac ultrasound in arrhythmogenic right ventricular dysplasia/cardiomyopathy: Two case reports

**DOI:** 10.1097/MD.0000000000019817

**Published:** 2020-04-10

**Authors:** Gabriel Cismaru, Alin Grosu, Sabina Istratoaie, Laura Mada, Maria Ilea, Gabriel Gusetu, Dumitru Zdrenghea, Dana Pop, Radu Rosu

**Affiliations:** a 5th Department of Internal Medicine, Cardiology-Rehabilitation; b 5th Department of Internal Medicine, Cardiology-Medical Clinic No5, “Iuliu Hatieganu” University of Medicine and Pharmacy, Cluj-Napoca; c Alba County Hospital, Department of Cardiology, Alba-Iulia, Romania.

**Keywords:** arrhythmogenic right ventricular cardiomyopathy, intracardiac ultrasound, thrombus, transesophageal echocardiography, ventricular tachycardia

## Abstract

**Rationale:**

Two-dimensional echocardiography (2D echo) is a major tool for the diagnosis of Arrhythmogenic right ventricular dysplasia/cardiomyopathy (ARVD/C). However 2D echo can skip regional localized anomalies of the right ventricular wall. We aimed to determine whether transesophageal and intracardiac ultrasound can provide additional information, on the right ventricular abnormalities compared to 2D echo.

**Patient concerns:**

Case 1 is a 30-year-old patient that presented in the Emergency Department with multiple episodes of fast monomorphic ventricular tachycardia (VT) manifested by palpitations and diziness. Case 2 is a 65-year-old patient that also presented with episodes of ventircular tachycardia associated with low blood pressure.

**Diagnosis:**

Both patients had a clear diagnosis of arrhythmogenic right ventricular dysplasia/cardiomyopathy confirmed by cardiac magnetic resonance imaging.

**Intervention:**

In both patients transesophageal and intracardiac ultrasound was performed, which brought more information on the diagnosis of ARVD/C compared to transthoracic echocardiograpy.

**Outcomes:**

The first patient was implanted with an internal cardiac defibrillator and treated with Sotalol for VT recurrences. He presented episodes of VT during follow-up, treated with antitachycardia pacing. The second patient was implanted with an internal cardiac defibrillator and treated with Sotalol without any VT recurrence at 18 month-follow-up.

**Lessons:**

Transesophageal echocardiography and intracardiac echocardiography can provide additional information on small, focal structural abnormalities in patients with ARVD/C: bulges, saculations, aneurysms with or without associated thrombus, partial or complete loss of trabeculations and hypertrophy of the moderator band. These changes are particularly important in cases with “concealed” form of the disease in which no morphological abnormalities are evident in transthoracic echocardiograpy.

## 1 Introduction

Arrhythmogenic right ventricular dysplasia/cardiomyopathy (ARVD/C) is a structural impairment of the ventricular myocardium characterized by replacement of myocites by adipose and fibrous tissue. Tissue changes lead to areas of abnormal arrhythmogenic tissue, accountable for ventricular arrhythmias such as premature ventricular contractions, ventricular tachycardia (VT) and ventricular fibrillation. In the concealed phase patients might present with ventricular arrhythmias; however, structural abnormalities may be evident with transthoracic echocardiography (TTE) only after years.

Despite the fact that diagnostic criteria^[1]^ have been proposed based on electrocardiogram (ECG), structural, histopathological, genetic, and arrhythmic features, ARVC is often difficult to diagnose. The 2010 Task Force diagnostic criteria include^[2]^: structural abnormalities (which can be detected in echocardiography or cardiac magnetic resonance imaging [MRI]), tissue abnormalities (which can be confirmed by biopsy), depolarization and repolarization disorders (highlighted on electrocardiogram), ventricular arrhythmias (shown on electrocardiogram or Holter ECG) and family load (confirmed by genetic analysis). Two-dimensional echocardiography (2D echo), based on its availability, low cost and ease of performance is a major tool for the diagnosis of ARVC.^2^,^3^,^4^ However 2D echo can skip regional localized anomalies of the right ventricular wall like: minor ventricular bulges, focal aneurysms, localized thinning of the ventricular wall, hypertrophy of the moderator band or disappearance of right ventricular trabeculations.^3^,^4^,^5^ Thus the value of 2D echo, in the diagnosis of ARVD is limited.^4^,^5^,^6^

We aimed to determine whether transesophageal and intracardiac ultrasound can provide additional information, on the right ventricular abnormalities compared to 2D echo.

## 2 Case reports

### 2.1 Case no 1

**Figure 1:**
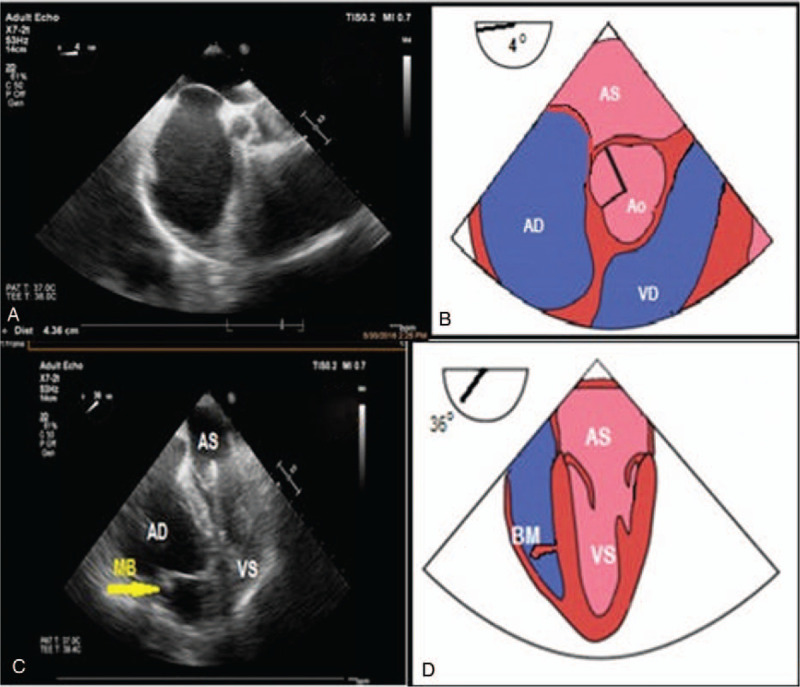
Transesophageal echography using a multiplanar probe. (A) Midesophageal view 4°: in the upper part of the image the left atrium can be seen- this is the closest anatomical structure to the esophagus. Separated by the interatrial septum, in the inferior part of the image lies the right atrium. The interatrial septum is convex towards the left atrium as the pressure inside the right atrium is high, given by the dilation of the right ventricle, tricuspid ring with subsequent tricuspid regurgitation. (B) Artwork with the structures seen in TEE. (C) Midesophageal view 36°: at the level of the right ventricle the moderator band (MB) can be visualized passing the ventricular cavity from the interventricular septum to the lateral wall where the anterior papillary muscle can be found. (D) Artwork with the moderator band as seen in TEE. TEE = transesophageal echocardiography.

A 30-year old patient presented to our Cardiology Department with several episodes of VT manifested by palpitations and dizziness. During VT episodes his blood pressure was 80/50 mm Hg therefore electrical cardioversion was performed. His ECG showed a left bundle branch block pattern of VT. One of the episodes associated cardiac arrest and was treated by electrical defibrillation. Echocardiography showed a dilated right ventricle (RV) and right ventricular outflow tract (RVOT) with tricuspid regurgitation grade 3. Cardiac MRI confirmed ARVD and an internal cardiac defibrillator (ICD) was implanted for secondary prevention of sudden cardiac death. Transesophageal echocardiography (TEE) was performed and confirmed the findings of TTE providing excellent visualization of the dilated and hypokinetic RV. TEE also described a localized RVOT aneurysm which was not clearly seen with TTE (Fig. 1).

Intracardiac echocardiography (ICE) was performed using a rotational probe inserted in the RV through the right femoral vein.^[7]^ The RV measured >48mm in diameter. The RVOT was also dilated >32 mm in diameter. The ICE and TEE probes could also assess the presence and degree of the tricuspid regurgitation. TEE and ICE focused on the lateral wall of the RV could also describe absence of trabeculations which is also a marker of ARVD/C. An ICD was implanted and Sotalol treatment was initiated because of VT recurrences under Amiodarone. The patient provided informed consent for publication of the case.

### 2.2 Case no 2

**Figure 2:**
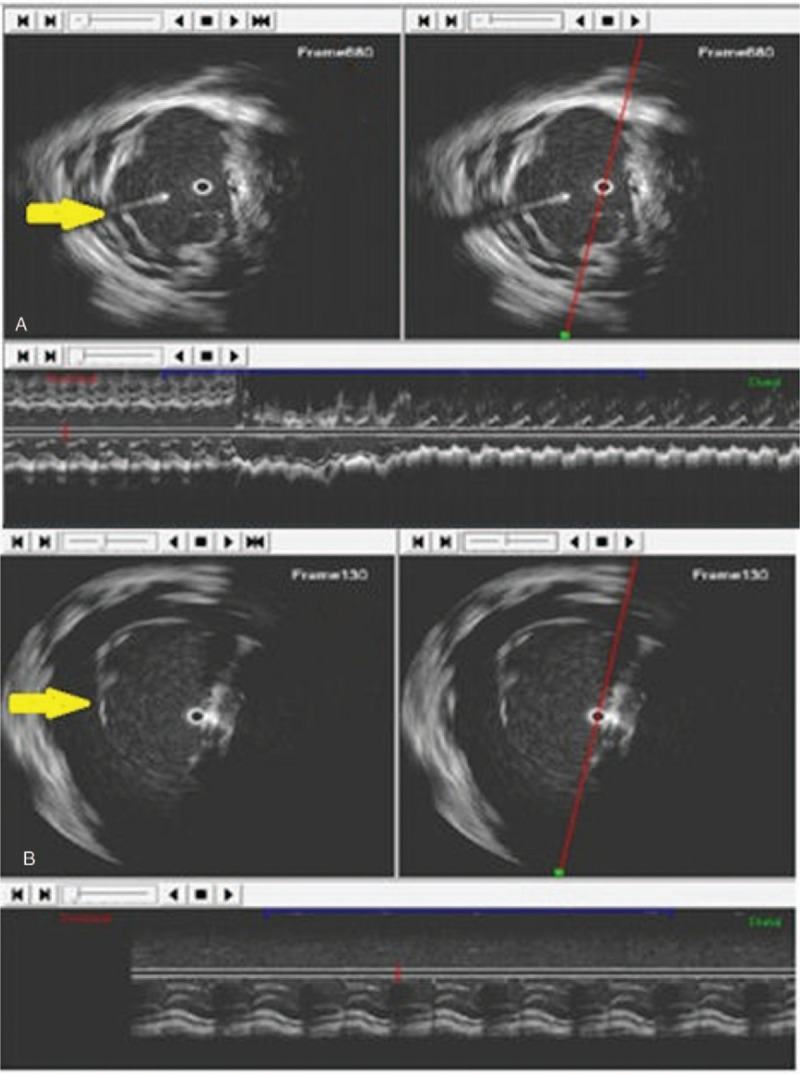
Intracardiac echography using a rotational probe. (A) The ViewFlex probe is inserted near the apex of the right ventricle. Please note the normal trabeculations which are present at the level of the lateral wall (yellow arrow). (B) The ViewFlex probe is inserted between the tricuspid valve and the apex of right ventricle. Please note the absence of normal trabeculations at the level of the lateral wall (yellow arrow). The regional absence of the trabeculations at the level of the “triangle of dysplasia” could not be seen with 2D echo and transesophageal echocardiography. 2D = 2-dimensional.

**Figure 3:**
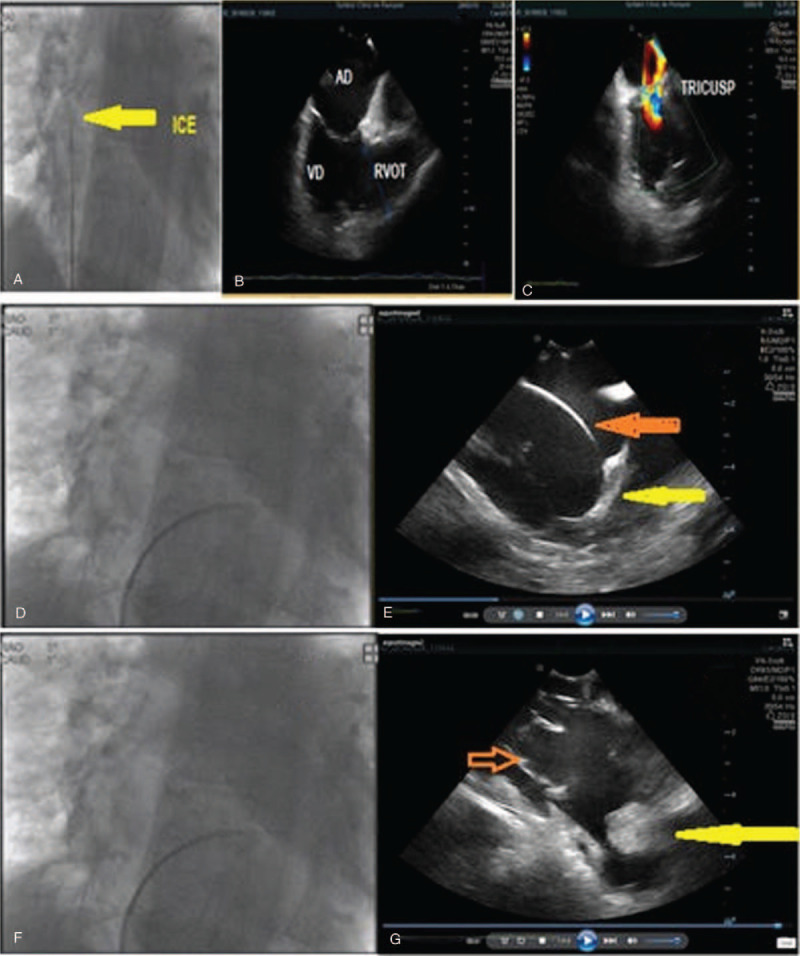
Intracardiac echocardiography using a sectorial probe. (A) Chest X-ray: the ICE probe is inserted in the middle of the right atrium. (B) The RA (44 mm), RV (52 mm), and RVOT (48 mm) are dilated. (C) As the tricuspid ring is dilated, a moderate tricuspid regurgitation is present. (D) Chest X-ray: Intracardiac echography with the probe inserted inside the right ventricle. (E) Near the apex of the right ventricle the moderator band can be visualized (yellow arrow). (F) Chest X-ray: Intracardiac echography with the probe inserted inside the right ventricle. (G) The patient is implanted with an ICD (red arrow) for the secondary prevention of sudden cardiac death as he presented many episodes of ventricular tachycardia. At the apex of the right ventricle a localized aneurysm can be seen with a pediculated thrombus (yellow arrow). ICD = internal cardiac defibrillator, ICE = intracardiac echocardiography, RV = right ventricle, RVOT = right ventricular outflow tract.

A 60-year old patient presented to our Cardiology Department with 2 episodes of VT with low blood pressure. Both episodes were stopped by electrical cardioversion. ECG showed left bundle branch block pattern VT. Echocardiography revealed a dilated RV with grade 4 tricuspid regurgitation. TEE showed an aneurysm of the RV apex with thrombus at this level which was further confirmed by cardiac MRI. RV wall thinning of less than 3 mm at the apical region was described with ICE. The thrombus was pedunculated, fixed in the aneurysmal region, at the apex of the RV. Bulges of the right ventricular free wall were present, with ICE being superior to TEE for their visualization (Figs. 1 and 3). Furthermore, a hypertrophy of the moderator band could be detected by TEE and ICE. For TEE we used the medioesophageal 4-chamber view and we scanned the chambers from 0° to 40° searching for the moderator band which crossed the RV cavity, passing from the interventricular septum to the anterior papillary muscle. From the transgastric view moderator band appeared like a thick and long band-like structure. ICE could give further information on the shape and structure of the moderator band: the septal attachment of the band was prominent with no branching and the papillary side showed branching with multiple connections to the anterior papillary muscle. The anterior papillary muscle was the most prominent of the 3 papillary muscles, received myocardial bundles form the moderator band and provided in its turn chordae to the anterior and posterior leaflets of the tricuspid valve (Fig. 2). The length of the moderator band was close to 30 mm with a thickness of >8 mm. The patient was discharged with Sotalol as Amiodarone was ineffective in controlling VT recurrences. An ICD was implanted, without any further VT episode during 18 months of follow-up. The patient provided informed consent for publication of the case.

## 3 Discussion

Four stages of ARVD/C were described:

(1) concealed phase (with no clinical manifestations of AVRD/C, but a potential risk of sudden cardiac death);
(2) overt electrical disease (with symptomatic arrhythmias);
(3) right heart failure; and
(4) biventricular failure.^[8]^

The clinician should be aware that ARVD/C cannot be excluded by the absence of structural abnormalities in TTE. In the concealed phase, ventricular arrhythmias often occur and structural abnormalities may be evident in TTE only after years. However, activation delay^[9]^ and prolonged electromechanical interval^[10]^ of the RV are present in concealed phases. In patients with concealed forms of ARVD/C, an electrophysiological study might induce VT during programmed ventricular stimulation.^[11]^ In both our patients with overt ARVD/C we performed an electrophysiological study and induced monomorphic sustained VT.

Echocardiography, widespread available, with low cost and easy to perform by residents and young specialists, plays an important role in the management of patients with suspected ARVD/C. Old echocardiographic criteria which included regional or segmental dilation, RV wall thinning, reduction of RV systolic function and formation of RV aneurysms were subjective and qualitative. The new 2010 revised Task Force diagnostic criteria include specific measurements of RV outflow diameter which is a more powerful criteria. TEE and ICE could help make a clear diagnosis of ARVD in stage 1 when no morphological abnormalities can be seen in TTE.

Our report shows that ICE and TEE may show abnormalities that are not evident in TTE. ICE and multiplanar TEE^[12]^ are comparable to MRI for describing structural abnormalities such as: localized RV or RVOT aneurysms,^[13]^ saculations and bulges, structures which may be missed by TTE. The importance of this localized structural abnormalities is that they can be sources of re-entry circuits for VT sometimes contributing to incessant forms of arrhythmia.

Confirmation of ARVD/C by TEE or ICE^[14]^ in such concealed forms provides an unfavorable prognosis given the aritmogenic risk of sudden cardiac death and disease progression to RV dilation with cardiac failure.

Moderator band hypertrophy is not a major or minor Task Force criteria for ARVD/C but often patients who die with the disease have hypertrophy described in autopsy. The moderator band or the septomarginalis trabecula is a muscle bundle that passes from the lower part of the interventricular septum to the lower part of the antero-papillary muscle crossing the RV cavity. Hypertrophy is very frequent in patients with ARVD/C. It occurs as a result of fat and fibrous cell infiltration, with penetration of the fibroadipose into the myocardium of the band, without a clear demarcation between the healthy and abnormal tissue. At this level lies the right branch of the conduction system, with Purkinje fibers at the end.

Despite the presence of RV dilatation, dysfunction and aneurysms with a relative blood stasis in patients with ARVD/C, reports of RV mural thrombi are rare.^[15]^ In our patient the heterogenous echogenicity and the laminated appearance in ICE, as well as the site of attachment to a thin RV wall, suggested it to be a thrombus. Cardiac MRI confirmed the thrombus and differentiated it from myxoma and other cardiac tumors. What is particular for ARVD/C and has been revealed in case reports of patients with thrombi, is that they often have a pedicle^[15]^ that attach to the ventricular endocardium. This was also the case of our patient who presented a pedunculated thrombus, the difference with a tumor being achieved with the help of gadolinium contrast. Our patient presented no symptoms or signs of pulmonary embolism. The prevalence of thromboembolic complications in patients with ARVD/C is approximately 4% over a follow-up period of over 8 years.^[16]^ The first manifestations of ARVD/C is frequently cerebral or pulmonary embolism, (including a pulmonary thromboembolism with sudden cardiac death, which has been recently reported.

In conclusion TEE and ICE can provide additional information on small, focal structural abnormalities in patients with ARVD/C: bulges, saculations, aneurysms with or without associated thrombus, partial or complete loss of trabeculations and hypertrophy of the moderator band. These changes are particularly important in cases with “concealed” form of the disease in which no morphological abnormalities are evident in TTE.

## Author contributions

**Investigation:** Alin Grosu, Laura Mada, Maria Ilea.

**Resources:** Gabriel Cismaru, Radu Rosu.

**Supervision:** Dumitru Zdrenghea, Dana Pop.

**Validation:** Alin Grosu, Maria Ilea.

**Visualization:** Gabriel Cismaru, Alin Grosu, Sabina Istratoaie, Laura Mada, Gabriel Gusetu.

**Writing – original draft:** Gabriel Cismaru, Alin Grosu, Sabina Istratoaie, Gabriel Gusetu, Radu Rosu.

**Writing – review and editing:** Gabriel Cismaru, Sabina Istratoaie, Dumitru Zdrenghea, Dana Pop, Radu Rosu.

Gabriel Cismaru orcid: 0000-0002-7352-9584.
